# Global Trends and Factors Associated with the Illegal Killing of Elephants: A Hierarchical Bayesian Analysis of Carcass Encounter Data

**DOI:** 10.1371/journal.pone.0024165

**Published:** 2011-09-02

**Authors:** Robert W. Burn, Fiona M. Underwood, Julian Blanc

**Affiliations:** 1 Department of Mathematics and Statistics, School of Mathematical and Physical Sciences, University of Reading, Reading, United Kingdom; 2 Monitoring the Illegal Killing of Elephants, CITES Secretariat, UNEP/DELC, Nairobi, Kenya; University of Western Ontario, Canada

## Abstract

Elephant poaching and the ivory trade remain high on the agenda at meetings of the Convention on International Trade in Endangered Species of Wild Fauna and Flora (CITES). Well-informed debates require robust estimates of trends, the spatial distribution of poaching, and drivers of poaching. We present an analysis of trends and drivers of an indicator of elephant poaching of all elephant species. The site-based monitoring system known as Monitoring the Illegal Killing of Elephants (MIKE), set up by the 10^th^ Conference of the Parties of CITES in 1997, produces carcass encounter data reported mainly by anti-poaching patrols. Data analyzed were site by year totals of 6,337 carcasses from 66 sites in Africa and Asia from 2002–2009. Analysis of these observational data is a serious challenge to traditional statistical methods because of the opportunistic and non-random nature of patrols, and the heterogeneity across sites. Adopting a Bayesian hierarchical modeling approach, we used the proportion of carcasses that were illegally killed (PIKE) as a poaching index, to estimate the trend and the effects of site- and country-level factors associated with poaching. Important drivers of illegal killing that emerged at country level were poor governance and low levels of human development, and at site level, forest cover and area of the site in regions where human population density is low. After a drop from 2002, PIKE remained fairly constant from 2003 until 2006, after which it increased until 2008. The results for 2009 indicate a decline. Sites with PIKE ranging from the lowest to the highest were identified. The results of the analysis provide a sound information base for scientific evidence-based decision making in the CITES process.

## Introduction

In spite of the ban on trade in ivory since 1990 there is continuing widespread concern about the illicit ivory trade and the illegal killing of elephants, both of which, to judge from press reports, are evidently still with us. The ban was imposed by the Convention on International Trade in Endangered Species of Wild Fauna and Flora (CITES) in the 7^th^ Conference of the Parties to CITES (CoP). In 1997 at the 10^th^ CoP, three countries in Southern Africa (Botswana, Namibia and Zimbabwe) successfully argued that “some of their elephant populations were healthy and well-managed” and that “income from limited ivory sales would bring benefits to conservation and to local communities” (CITES Press Release: http://www.cites.org/eng/news/press/2007/070228_cop14.shtml). Habitat degradation [1] and human-elephant conflict [2] are often cited as consequences of “locally overabundant” elephant populations in Southern Africa. The CoP agreed to a change in CITES listing of the African elephant from CITES Appendix I to Appendix II for the three countries, and the down-listing was in 2000 extended to include South Africa also. This decision and the consequent licensed one-off sales of ivory have proved to be controversial and there is no sign that the debates around these issues are subsiding. Indeed, in successive CoPs since the down-listing was agreed, more time has been spent discussing African elephant issues than any other single species [3].

The central issues upon which much of the current debate is focused are: (1) Is there a trend in elephant poaching and if so, how strong is it? (2) Have changes in CITES policy, and in particular the one-off ivory sales, had an impact on elephant poaching? Debates in successive CoPs have tended towards a polarization of views. One side contends that any relaxation of restrictions on trade in ivory amounts to a green light to poachers and that any perceived increase in poaching must be attributable to it. The opposing view argues that there are many factors that could potentially explain an increase, and that CITES listings cannot be assumed to be of any great interest to the poaching fraternity. To judge from the debates that have taken place, it appears that a sound evidence base, in support of either viewpoint, is lacking. What studies there have been have either been of limited geographical scope [4], [5], or have addressed the occurrence of poaching only indirectly by inferring it from changes in elephant abundance estimates or overall mortality [4]–[8]. Analysis of time trends in poaching has been restricted mostly to graphical displays of population or mortality data, or a simple linear trend.

The question of an association between CITES policy and trends in illegal ivory trade cannot be considered in isolation. There are many potential drivers of the illegal killing of elephants and it is necessary to situate the impact of CITES policy within a broader causal framework. Existing studies that have addressed the question (e.g. [6]) have sought direct statistical evidence of an effect of one-off sales without addressing the issue of other, potentially confounding, effects. Other authors [9] appear to take the relationship as self-evident without the need for any data analysis at all. We contend that a prerequisite for measuring the impact of CITES policy must be to assemble data on all potential associated factors and to assess not only their effect on poaching, but also the inter-relationships between them (http://www.cites.org/common/prog/mike/data/data_analysis_strategy.pdf). These factors may include both proximate causes (e.g. ease of access to the elephant population, or site-level law enforcement effectiveness) and ultimate causes, such as economic factors and governance. Until we have a reasonably complete picture of the overall causal backdrop, it will be impossible to address the question of the relationship between CITES policy and illegal killing in a meaningful way.

CITES is a global treaty and assessing the impact of its decisions is best attempted at a global level. This has hitherto been difficult owing to the lack of data on illegal killing of elephants across the elephant's range, but data are now becoming available. A condition for the 1997 partial down-listing of the African elephant was the establishment of two global monitoring systems: Monitoring the Illegal Killing of Elephants (MIKE) and the Elephant Trade Information System (ETIS) (www.cites.org/eng/res/all/10/E10-10R15.pdf). The objectives of these systems include tracking trends in illegal elephant hunting and trade in ivory, and assessing the extent to which changes in these trends are related to CITES decisions on elephants. MIKE has focused data collection exercises in selected sites in Africa and Asia. MIKE data from these sites include elephant population surveys and data collected by anti-poaching patrols and other sources. The patrol data include records of elephant carcass encounters – cause of death (specifically, whether the elephant was illegally killed or died from other causes), and the estimated age of the carcass. Data that are available uniformly across all MIKE sites are site by year aggregates of carcass encounters and the number of these that were illegally killed. These data provide the first opportunity to consider trends in illegal killing across Africa and Asia and a context in which drivers of illegal killing can be considered at global, national and site level.

Analysis of these data entails a number of limitations that need to be borne in mind. First, in spite of early efforts to achieve a representative selection of sites [http://www.cites.org/eng/prog/MIKE/intro/index.shtml], the sites currently covered by MIKE cannot claim to be a truly random sample of sites from all elephant range areas. The second concern is a general difficulty in the analysis of law enforcement patrol data. Patrols are variable, with big differences in, for example, the distance covered and the intensity of patrolling, resulting in variability in the chance of encountering a carcass. This can be true even at a single site and is compounded when considering different sites with varying resources, habitats and conditions. Standardizing across patrols is conventionally achieved by using a measure of patrol effort with which some sort of catch per unit effort analysis (CPUE) can be performed [10]. However, obtaining robust measures of law enforcement effort that are applicable across all MIKE sites has so far turned out to be problematic. Reporting of patrol effort is one aspect of MIKE data that has so far been particularly uneven, and effort data at the level of detail of individual patrols is very patchy. A third limitation derives from the inevitable heterogeneity between sites. This arises partly because of variations in the type of patrolling that is used, and also because of widely differing resources across MIKE sites, which encompass sites in southern Africa and Asia relatively rich in resources, and sites in remote forest areas in central Africa suffering from current or recent civil strife.

Despite these limitations, MIKE carcass encounter data provides a rich source of data on illegal killing of elephants from across the entire range of African and Asian elephants. We present the first analysis of carcass data from 66 MIKE sites over the period 2002–2009. Our aims were to

1. describe trends in the illegal killing of elephants over time;
2. identify site- and country-level factors associated with illegal killing of elephants;
3. describe and compare rates of illegal killing of elephants across sites and range states.

We avoid the difficulty of not having reliable patrol effort data by using the *proportion of illegally killed elephants* (PIKE*)* – defined as the ratio of number of carcasses illegally killed to the total number of carcasses encountered – as a relative index of illegal killing. We assume that this measure is more or less independent of effort (although potential sources of bias are described in the Discussion). We identify a number of potential factors associated with illegal killing and investigate their effect on PIKE. Some factors are measured at country level whilst others are recorded at site level and we account for this in the analysis by fitting hierarchical models. These site-level and country-level covariates do not attempt to explain the trend through time but to identify reasons why PIKE differs between sites and countries. Because of the non-random nature of the data we have chosen not to carry out formal statistical testing and instead use a measure of the strength of evidence for comparing statistical models [11] and identifying important factors. Furthermore, we have adopted a Bayesian approach [12] to better represent the uncertainties in the data and in the models. Our analysis enables us to describe non-linear trends in illegal killing of elephants through time and provides the first contribution to the identification of site or country level drivers of the illegal killing of elephants.

## Materials and Methods

### Data

The data were derived from 6,337 carcasses of elephants encountered by patrols in 66 MIKE sites in 36 range states in Africa and Asia between 2002 and 2009. This was the dataset remaining after removing three sites (Kahuzi Biega in the Democratic Republic of Congo, Bukit Barisan Selatan in Indonesia and Gua Musang in Malaysia) where no carcasses were recorded in any year. The distribution of the sites across the elephant range is shown separately for Africa and Asia in Figures S1 and S2.

For each carcass, cause of death was classified as illegal or not, and year of death was assigned according to standard carcass ageing criteria [13]. The data analysed were site by year totals of number of carcasses encountered and number of illegally killed carcasses. These totals are in Table S1. A blank in a site year cell indicates either that no data were provided by the site in that year or that no carcasses were found on patrol; the analysis does not need to distinguish between these situations.

We used the proportion of illegally killed elephants (PIKE) among the carcasses encountered by patrols as an indicator of poaching. The population parameter corresponding to this statistic is the probability that an elephant carcass was illegally killed. This is a relative measure and is not the proportion of elephants in the population that have been illegally killed – this cannot be estimated with the available data. The use of PIKE appears to sidestep the need for a measure of effort because we assumed that in the PIKE ratio, effort appears in both numerator and denominator and effectively “cancels out”. The simplification does not come free, however, and we critically examine the implicit underlying assumptions in the Discussion section below. A bonus of an effort-free method of analysis is that we can accommodate sites with different types of patrol which would require qualitatively different measures of effort. The “beat” system commonly used in India [14] is very different from the patrol regime used in most of Africa. One African site with completely different carcass encounter data is Samburu-Laikipia in Kenya [15], where the data are not derived from patrols at all, but from a system based on a network of informants. These can all be accommodated in an analysis based on PIKE.

We were guided in the choice of candidate covariates by the aims of the analysis, in particular to enable characterization of sites and countries with high levels of elephant poaching, and to contribute towards an understanding of its general causal background. Variables were selected on the basis of prior expectation of relevance to illegal killing. Site-level covariates included in the analysis are listed in Table 1. Site characteristics represented by these variables were: the size of the site, *area*, the size, *ele*, and density, *dens*, of the elephant population, ecosystem type, *ecosys*, human population, *pop*, human pressures in and around the site, *ftprint*, and conservation effort, *conseff*. Ecosystem type was measured by a continuous variable, the net primary production of the site. High values represent sites with more forest cover and exploratory analysis indicated a strong correlation between high forest cover and net primary production for sites in Africa. There was no available data on forest cover for sites in Asia hence our use of net primary production as a proxy. Conservation effort was measured by a proxy variable – the probable fraction – which is a measure of the precision of elephant population estimates cited in the African Elephant Database [16]. Factors that determine the precision include the resources available to the survey teams, and thus the probable fraction can be interpreted as a proxy for conservation effort with higher values indicating greater effort. High values may, in some cases, reflect the fact that external NGOs have carried out the elephant population survey rather than conservation effort devoted to the site by the government. However, as NGOs also tend to devote resources to law enforcement and infrastructure management our variable may still be a reasonable reflection of conservation effort at that site. The UNEP-WCMC programme on protected areas (http://www.unep-wcmc.org/protected-areas_24.html), has an alternative measure on conservation effectiveness, but we had difficulty assembling enough data to get a reasonable coverage of the sites used in our study. Specific details of how the site-level variables were obtained are given in Text S1 and the data are provided in Table S2.

**Table 1:** Site-level covariates.

Name	Description	Source
*area*	Area of site (km^2^)	AED^a^
*ele*	Estimated size of elephant population	AED^a^ & elephant surveys
*dens*	Estimated elephant density	Derived from *area* and *est*
*ecosys*	Net primary production (see text)	Imhoff et al, 2004 – CIESIN^b^
*people*	Human population density	LandScan™, 2006
*pop*	= 1 if *people* >100, = 0 otherwise	Derived from *people*
*ftprint*	Human footprint (see text)	WCS^c^ & CIESIN^b^, 2002
*conseff*	Conservation Effort (see text)	AED^a^ & elephant surveys

a AED: African Elephant Database.

b CIESIN: Centre for International Earth Science Information Network.

c WCS: Wildlife Conservation Society.

The variables *area*, *ele*, *dens* and *people* all had positively skew distributions and were therefore replaced by their natural logarithms in the analyses. For model fitting, the variable ln(*people*) was still right skew with a small number of sites with very high density. Furthermore, these sites, although they had very low numbers of carcasses, were found to have a very high influence on the fitted models. The variable was therefore replaced by a binary categorical variable *pop* defined as
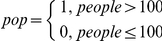
Of the 66 sites in the study, 15 were in the high population density group.

Country-level covariates were chosen to represent aspects of governance, demographic change, the economy and human development. The variables used are summarized in Table 2 and data are in Table S3. Measures of governance were obtained from the World Bank's *World Governance Indicators (WGI)* project (http://info.worldbank.org/governance/wgi/index.asp). These variables were supplemented by the Corruption Perceptions Index (CPI) from Transparency International (http://www.transparency.org/). We included CPI because it is the index of corruption that has been most widely used in previous studies of conservation and biodiversity [8], [17]. Countries with large values of these variables have better governance. Basic demographic and economic measures were obtained from the United Nations Statistics Division (UNSD) (http://unstats.un.org/unsd/). We used the human development index produced by the United Nations Development Programme (UNDP) (http://hdr.undp.org/en/statistics/). This is a composite index derived from measures of educational attainment, human life expectancy and income. The first two of these variables were also included in the set of country-level covariates. Finally, a measure of domestic ivory market activity was included in the covariates. This is a measure produced by MIKE's sister project, the Elephant Trade Monitoring System (ETIS) for its analysis of illegal ivory seizures data (http://www.cites.org/common/cop/13/inf/E13-29-2A.pdf gives details of the calculation). We included it here because we thought that it was possible that unregulated domestic ivory markets might impact on the local intensity of elephant poaching. Large values represent countries with bigger domestic ivory markets.

**Table 2:** Country-level covariates.

Name	Description	Source
*ConCorr*	Control of corruption	World Bank
*GovEff*	Government effectiveness	World Bank
*PolStab*	Political stability and absence of violence	World Bank
*RuleLaw*	Rule of law	World Bank
*RegQual*	Regulatory quality	World Bank
*VoicAcc*	Voice and accountability	World Bank
*CorrPI*	Corruption perceptions index	Transparency International
*GDP*	Gross domestic product per capita	UNDP^a^
*PopGth*	Annual population growth rate	UNSD^b^
*ODAid*	Overseas development aid per capita	UNSD^b^
*EduAtt*	Educational attainment	UNDP^a^
*LifeExp*	Human life expectancy	UNDP^a^
*HDevI*	Human development index	UNDP^a^
*DomIvry*	Index of domestic ivory markets	ETIS^c^

a UNDP: United Nations Development Programme.

b UNSD: United Nations Statistics Division.

c ETIS: Elephant Trade Information System.

Data for both site- and country-level covariates were available over varying ranges of years, and in some cases only for one or two years. To overcome this, and to simplify the analysis somewhat, we took only the 2007 values of all variables. Preliminary analysis indicated that there was much more variability in the values of the covariates between countries and between sites than between the relatively short span of years covered by the data.

### Statistical Methods

Before embarking on statistical modeling of PIKE, the covariates were subjected to preliminary exploratory analyses, separately at site and country levels. The aim was to understand the inter-relationships among covariates to aid in the selection of variables and the interpretation of the final models. For this we used principal components analysis (PCA), and obtained visualizations of the results using plots of the loadings of the first two principal components [18].

The analysis of PIKE was based on fitting statistical models to the data. The basic statistical modeling tool used was hierarchical binomial logistic regression [19], with three levels: countries, sites within countries and years within sites, to capture the data structure. All covariates were fitted as fixed effects – i.e. with constant regression coefficients across sites and countries. We considered the possibility of a non-linear time trend by fitting orthogonal polynomials up to order 7, the maximum possible with eight years of data. Models were fitted in a Bayesian framework in order to take full account of all sources of uncertainties. Details of the benefits of fitting Bayesian hierarchical models can be found in Text S2. We use the following notation to describe the models fitted:

*n_ijk_* = number of carcasses found in year *i* = 2002, …, 2009, site *j* = 1, …, *m_k_*, country *k*.
*y_ijk_* = number of illegally killed carcasses encountered, 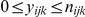

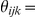
probability that a carcass was illegally killed (the PIKE parameter).

Specifically, the fitted models were of the general form
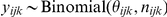


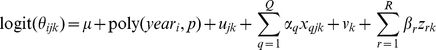
where 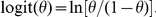 The terms *u_jk_* and *v_k_* are site- and country-level deviations (random effects) from the intercept, *μ*, and are assumed to have independent normal distributions 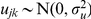 and 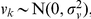 respectively. Orthogonal polynomial terms for the time trend are represented by poly(*year_i_*, *p*) where *p* is the order of the polynomial. The *x_qjk_*, are site-level variables and the *z_rk_*, country-level variables, all standardized to have a mean of zero and a standard deviation of one.

The modeling strategy was as follows. First, a model with random intercepts for countries and sites within countries only was fitted, with no covariates. This was the minimal model, in the sense that it represented just the hierarchical structure of the data, without covariate effects. Next we added a polynomial function of year while at the same time determining the best fitting order of polynomial. An initial exploratory analysis of time trend, providing an idea of the polynomial order to expect, was accomplished by fitting a cubic spline smoother (using generalized additive models [20]). This model, representing data structure plus polynomial time trend, was taken as the baseline model to which covariates were added to estimate their effects. We explored combinations of site-level covariate, including interactions between them, that best explained the data. Then, having settled on site-level variables, country-level variables were fitted in a similar way to get the best fitting combination. The reason for choosing to fit site-level variables first was that these were likely to represent proximate causal effects, having more immediate effects on poaching than country-level variables. Having included country-level covariates that seemed important in the model, the site-level variables were re-tested in case their relative influence had changed. In principle, this process can be repeated iteratively until a stable choice of covariates emerged, but in the event, no further iterations were needed. First-order interactions between site and country level variables in the model were then considered. Choices of country- or site-level covariates were guided by the results of the PCAs so that if a group of covariates were highly correlated each was assessed and the most important included in the model.

Non-informative priors were used throughout. Specifically, these were as follows.



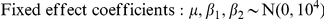


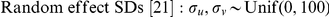



We took the view that there was no statistical basis for using the conventional null hypothesis testing approach to model selection: the data are purely observational, with no means of controlling for unwanted sources of variability as would be expected in a controlled experiment [22]. What is more, the site selection process was non-random, and most of the patrol data were obtained from non-random, and sometimes purposive, sampling. These features combine to threaten even the most liberal interpretation of the underlying assumptions required of formal statistical test procedures. Instead we used the Akaike Information Criterion (AIC) to compare different models and determine the important variables, whilst allowing the possibility of a multi-model conclusion [11]. Having fitted a set of candidate models, the AIC weights (calculated from a comparison of each model's AIC with that of the lowest AIC) were computed. These weights can be interpreted as the relative weight of evidence in favour of each of the candidate models. Initial model exploration and fitting was undertaken in a frequentist (i.e. non-Bayesian) framework and AIC values were obtained from these models. Although the deviance information criterion (DIC) has become popular for Bayesian modeling, there are situations where AIC should be used instead (the reasons concern the *focus* of the inference in hierarchical models [Spiegelhalter DJ: http://www.mrc-bsu.cam.ac.uk/bugs/winbugs/DIC-slides.pdf]).

Having fitted models to the data, we used the MCMC simulations to obtain predicted values of PIKE. The use of model predictions for inferences about PIKE is tantamount to using smoothed values rather than simply calculating the raw proportions directly from the data. The random “noise” in the raw data, not accounted for by the covariates, was summarized in the random effects, or residuals, at site and country levels.

## Results

### Data Coverage

Table S1 shows the number of carcasses found at each site in each year and the numbers of these carcasses that were illegally killed. Table 3 provides summaries of number of carcasses and PIKE for each sub-region and year.

**Table 3:** Proportion of illegally killed elephants (with numbers of all carcasses encountered) by year and sub-region.

	Region						
Year	Central Africa	Eastern Africa	SouthernAfrica	West Africa	Asia	Total	
**2002**	0.00 (5)	0.36 (165)	0.19 (53)	0.12 (17)	- (-)	**0.30 (240)**	
**2003**	0.70 (269)	0.25 (336)	0.11 (115)	0.24 (21)	0.08 (12)	**0.39 (753)**	
**2004**	0.79 (383)	0.33 (259)	0.21 (165)	0.35 (34)	0.05 (40)	**0.49 (881)**	
**2005**	0.54 (229)	0.23 (243)	0.06 (247)	0.30 (10)	0.12 (69)	**0.26 (798)**	
**2006**	0.63 (126)	0.22 (239)	0.19 (240)	0.00 (4)	0.18 (17)	**0.29 (626)**	
**2007**	0.87 (241)	0.32 (288)	0.16 (200)	0.78 (18)	0.03 (33)	**0.44 (780)**	
**2008**	0.86 (220)	0.50 (495)	0.22 (202)	0.86 (22)	0.09 (35)	**0.51 (974)**	
**2009**	0.64 (101)	0.29 (952)	0.31 (163)	0.86 (35)	0.50 (34)	**0.34 (1285)**	
**Total**	**0.74 (1574)**	**0.32 (2977)**	**0.17 (1385)**	**0.53 (161)**	**0.15 (240)**	**0.39 (6337)**	

There is considerable variability in the numbers of carcasses reported, both between sub-region and between sites within sub-regions and through time. Eastern Africa recorded twice as many carcasses as Southern and Central Africa and more than ten times as many carcasses as West Africa and Asia. Large numbers of carcasses were found at sites with large elephant populations and West African and Asian elephant populations are much smaller than in other sub-regions. Of the 2977 carcasses found in Eastern Africa about 50% (1529) were found at Samburu-Laikipia (SBR) in Kenya, collected using an informant network [15]. The low number of carcasses in 2002 is because many sites, including all those in Asia, were not yet reporting to MIKE.

### Exploratory Analysis of Covariates

Details of the results of the PCA analyses are provided in Text S3 and Figures S3 and S4 show the first two principal components for the country-level and site-level PCAs respectively. The key result for the country–level PCA is that there are two clear groupings of variables. These groups represent variables describing *governance* and variables describing *development*. Although these are distinct groups with high correlation between variables within these groups there is also correlation between the two groupings. In the statistical modeling, variables from the *governance* group were compared to determine the most important to retain in the model if appropriate and similarly for variables from the *development* group. With the site-level PCA the associations are less clear, although human footprint, *ftprint*, and human population, ln(*people*), are strongly associated and the area of the site, ln(*area*) is negatively correlated with both of them. The variable representing forest cover, *ecosys*, accounts for most of the 18.7% of the variation explained by the third principal component.

### Models for PIKE

The minimal model representing the data structure was a hierarchical logistic regression model with random effects for both sites and countries, and no covariates:
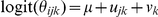
This model had AIC = 1199.5. For the time trend, a fifth-order polynomial was found to fit the data well (the AIC dropping to 1062.2):
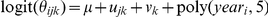
The estimated variation between sites within countries (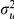) was 1.34 and the estimated variance between countries (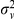) was 2.50 so there was nearly twice as much variability between countries than between sites within countries.

**Figure 1:**
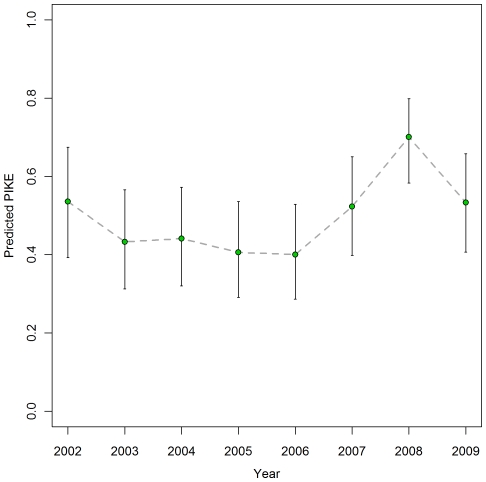
Trend in *PIKE* through time. Mean annual PIKE by year with 95% credible intervals.

The trend is shown graphically in Figure 1. This model was the baseline for assessing all subsequent models. Adding site and country-level variables to the model did not affect the form of the trend because these variables explain average differences between sites and countries and do not explain differences over time.

The variables that were found to be important were *ecosys*, *pop* and ln(*area*) at site level, and *GovEff* and *HDevI* at country level. Table 4 summarizes the fit, in terms of AIC and AIC weights, of all fitted models containing these covariates. The models are listed in order of decreasing AIC, not the order of fitting. It is apparent from Table 4 that *ecosys* is the most important site-level covariate and that the effect of ln(*area*) overall is negligible (comparing Models 7 and 8). The *area* effect becomes large, however, when considered separately for each level of *pop* (i.e. the *pop*×ln(*area*) interaction causes a substantial drop in AIC).

**Table 4:** Fixed effects terms of fitted models.

Model, *i*	Fixed effects	*AIC_i_*	*w_i_*
1	none	1199.5	0.0000
2	p(*year*,5)	1062.2	0.0000
3	p(*year*,5)+*ecosys*	1051.1	0.0000
4	p(*year*,5)+*ecosys*+*HDevI*	1044.2	0.0000
5	p(year,5)+*ecosys*+pop*ln(*area*)	1039.4	0.0002
6	p(*year*,5)+*ecosys*+*GovEff*+*HDevI*	1033.7	0.0036
7	p(*year*,5)+*ecosys*+*GovEff*+ln(*area*)	1033.5	0.0040
8	p(*year*,5)+*ecosys*+*GovEff*	1033.4	0.0042
9	p(*year*,5)+*ecosys*+*GovEff*+pop*ln(*area*)	1024.9	0.2958
10	p(*year*,5)+*ecosys*+*HDevI*+pop*ln(*area*)	1023.2	0.6921

All models have random effects for countries and sites within countries. The w_i_ column shows the AIC weights and p(year,5) is the polynomial of order 5 for the year effect.

The inference for country-level covariates is less clear. Both *GovEff* and *HDevI* have quite large effects, but the inclusion of either one of them in the model makes the other redundant. The relationship between these variables was noted in the plot (Figure S3) of the PCA for country-level variables (the correlation between them is in fact 0.64), so it is not surprising that they partially annihilate each other in fitted models. This ambivalence can be resolved by allowing multi-model inference. Although Model 10 was the best fit according to AIC, Models 9 and 10 between them have total AIC weight of 0.99. We therefore conclude that that data provide evidence that supports both *GovEff* and *HDevI* as having an effect. It should be noted that with mixed models, such as we have here, there are difficulties with the usual definition of AIC [11]. Although the AIC values in Table 4 can probably serve as a rough guide to model selection, more reliable inferences about particular model parameters are obtained from credible intervals in the Bayesian analysis, shown in Table 5. This table shows the posterior means of the parameters in models 9 and 10 – note that the values for the polynomial trend terms were virtually identical in the two models. The 95% credible intervals for these terms are all either entirely positive or entirely negative and are well clear of zero, indicating that the time trend can be regarded as important, or “significant”.

**Table 5:** Estimates of parameters in fitted Models 9 and 10 – posterior means and 95% credible intervals.

Model term		Posterior mean	Lower limit	Upper limit
**Models 9 & 10**				
p(*year*,5)	linear	3.95	2.75	5.17
	quadratic	2.47	1.20	3.75
	cubic	−3.24	−4.48	−1.99
	quartic	−3.31	−4.51	−2.12
	quintic	−2.83	−4.04	−1.61
**Model 9**				
*Site-level*	*ecosys*	0.64	0.25	1.06
	*pop*	−0.75	−2.09	0.60
	ln*(area) (pop = *0*)*	−0.68	−1.14	−0.23
	ln*(area) (pop = *1*)*	0.61	−0.49	1.77
	Variance 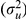	1.17	0.54	2.19
*Country-level*	*GovEff*	−0.98	−1.52	−0.49
	Variance 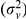	0.64	0.01	1.86
**Model 10**				
*Site-level*	*ecosys*	0.89	0.52	1.28
	*pop*	−0.98	−2.33	0.37
	ln*(area) (pop = *0*)*	−0.90	−1.37	−0.46
	ln*(area) (pop = *1*)*	0.53	−0.59	1.73
	Variance 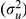	1.27	0.62	2.28
*Country-level*	*HDevI*	−1.10	−1.63	−0.60
	Variance 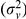	0.37	0.00	1.38

**Figure 2:**
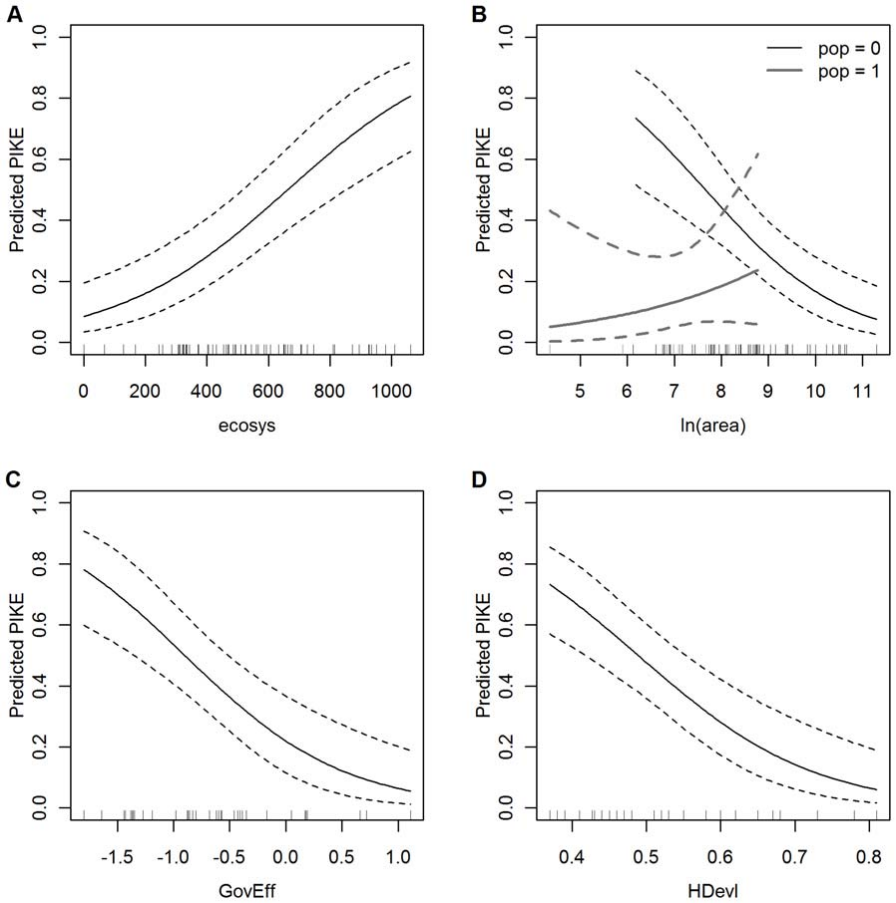
Predicted mean *PIKE* plotted against fitted covariates. Posterior mean of PIKE for varying (A) *ecosys* (B) ln(*area*) (C) *GovEff* and (D) *HDevI* with 95% credible intervals. All other covariates set to their mean values, *pop* = 0 unless shown and *year* = 2006. Rug plot at bottom of each graph shows data values for the relevant variable.

Further conclusions can be drawn from Table 5. Sites with higher *ecosys* tend to have higher PIKE – indicating a greater mean rate of poaching in forest sites than in savannah sites. Figure 2(A) shows this effect. Overall, the effect of human population density (*pop*) seems to be small (the credible interval straddles zero), but it is important when considering the area effect: at sites with low human population density (*pop* = 0), there is quite strong evidence that large sites (as measured by ln(*area*)) tend to have lower PIKE than smaller ones. The estimated relationship is shown in Figure 2(B). On the other hand there is no evidence of an *area* effect at sites with high human population density (*pop* = 1). There is clear evidence from Model 9 that governance, as measured by the *GovEff* (government effectiveness) variable, has a strong negative relationship to PIKE – i.e. PIKE tends to be lower in countries with good governance. This effect is shown graphically in Figure 2(C). Model 10 provides clear evidence of an *HDevI* effect – higher levels of human development tend to be associated with lower values of PIKE. This relationship is shown in Figure 2(D). In these two models the variance terms, 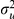 and 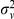, indicated that there was more “unexplained” variability (i.e. not accounted for by the covariates) between sites within countries than between countries. Without covariates there was more variability between countries than between sites within countries, so country-level covariates are explaining more of the differences between countries than the site-level covariates explain differences between sites within countries. Comparing the estimates for the site-level parameters between Models 9 and 10, we see that they are numerically somewhat different. However, because the estimates are contained within the credible interval of the other model the overall conclusions remain unchanged.

### Model Predictions for PIKE

**Figure 3:**
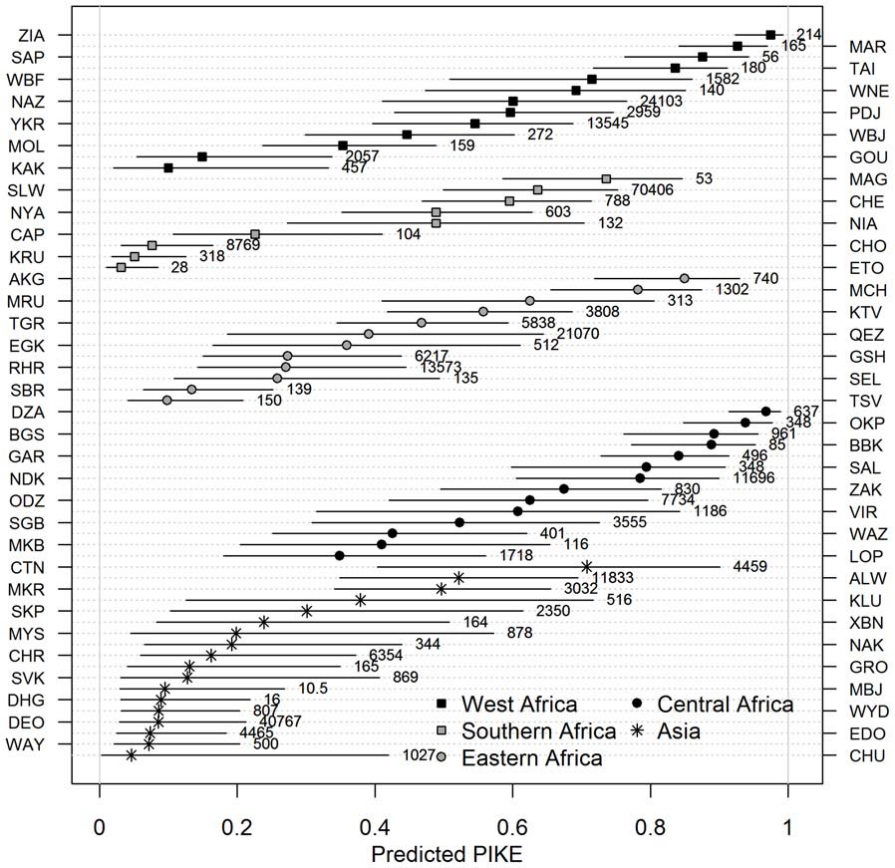
Predicted mean PIKE at each site for 2009. Posterior mean value of PIKE with 95% credible intervals. Numbers are estimated elephant abundances at each site. The names of the sites corresponding to the site codes shown on the vertical axis are given in Table S1.

For the purpose of comparisons among sites, the posterior predicted mean values of PIKE for each site (grouped by sub-region) in 2009 are shown in Figure 3. The line segments represent 95% credible intervals for the mean PIKE values. These predictions were derived from Model 10. Sites with small samples tend to have wide credible intervals.

The site- and country-level random effects (or residuals) are shown in Figures S5 and S6, respectively. Small values of the random effects indicate relatively small deviations of the observed values of PIKE from the values predicted by the model. Thus, the model appears to have performed reasonably well in Eastern Africa, and in most sites in Central and Southern Africa. We can deduce, however, that there are sites in West and Central Africa where PIKE appears to be considerable higher than predicted, suggesting that there are other factors associated with elephant poaching there. We also see that in most Asian sites, PIKE is generally lower than the predicted values.

## Discussion

### Trends over time

Referring to Figure 1, within the limits of uncertainty suggested by the 95% credible intervals, the trend in the annual mean value of PIKE over the period from 2002 until at least 2006 is relatively stable, although there is a slight suggestion of a decline after 2002. The following two years indicate a rise, followed by another decline in 2009. Care is needed in interpreting this trend. In particular, it is important to note that Figure 1 represents a global average value of PIKE, and that there is significant variation between sub-regional trends. Table 3 shows, for instance that although there is a decrease in the mean PIKE for both Central and Eastern Africa, this is not true for the other sub-regions: there is no change in West Africa while the Asia proportion increases. Note however, that some sub-regions are less well represented in the data than others, and their contribution to the global average is correspondingly diminished. Further reasons for exercising caution in interpreting the trend are based on potential biases in the PIKE statistic, discussed below.

### Factors associated with PIKE

Among site-level factors, *ecosys* was found to have a clear association with PIKE. This variable is a proxy for vegetation cover and the analysis indicates that sites with forest cover experience higher levels of poaching than the savannah sites, presumably because poachers have greater freedom of movement without detection by law enforcement officials. In particular, in Central Africa, where most sites have a tropical rainforest habitat, PIKE has tended to be high, as can be seen in Figure 3. Some evidence for the intensive poaching of Central African forest elephants in recent years has been documented elsewhere [4], although much of this evidence is indirect in the sense that it has been deduced from poacher signs or inferred from elephant population numbers and their distribution, rather than direct observation of elephant carcasses as in our data. Our analysis indicates that the size of a site, ln(*area*), is related to poaching, although only at sites where the human population density is low (Figure 2(B)). This finding is compatible with results presented by Blake et al [4]. At these sites, there is a clear tendency for PIKE to be higher in smaller sites. There is no such relationship, however, where the human population density is high. On the other hand, it could be argued that none of the very large sites are in densely populated regions, so with these data it has not been possible to properly test the influence of site area in those sites, if there is any. It is perhaps surprising that conservation effort at site level was not found to be associated with PIKE. However, the *conseff* variable used as a surrogate for conservation effort is probably not a good proxy and a more suitable measure needs to be found.

At country level, we conclude from the analysis that both governance and the level of human development are associated with PIKE, and that there is insufficient information in the data to reject one in favour of the other. It is not surprising to find these two aspects emerging jointly in our analysis – the relationship between governance and development has been researched extensively (see, for example, the “Governance and Development Review” published by the Institute of Development Studies at http://www2.ids.ac.uk/gdr/). Other studies [8], [17] have found statistical relationships between governance, or corruption, and conservation failures or biodiversity loss. These analyses have been criticised on both conceptual and statistical grounds [23]. The conceptual issue concerned how corruption is used to infer causality from an analysis which fails to account for the complex causal mechanisms that probably link it with conservation outcomes. We contend that there is a long history of establishing a statistical association before there was an understanding of the underlying causal mechanisms. In our current efforts to analyse data on illegal killing of elephants, we are only just beginning to make inroads into understanding the plethora of potential impacts – social, political, economic and ecological, and the causal pathways between them – on elephant poaching and illicit ivory trade. In the meantime, however, we hope that the associations between governance, development and illegal killing found in this study will contribute to an understanding of the complex backdrop of potentially causal factors, as well as being useful in themselves as indicating where to look for potential mitigating actions. Given that covariates explain much more of the variability in PIKE between countries than between sites within countries, even though there is more variability in PIKE between countries than between sites, more work is required to identify appropriate site-level covariates to explain the between site variability.

### Site comparisons

Because of the covert nature of poaching, it is clearly virtually impossible ever to devise an absolute measure of the rate of poaching based on direct observation. For the purposes of comparison across sites, however, we suggest that the predicted PIKE means, as presented in Figure 3, provide a reasonable relative index, subject to the limitations discussed below. It is important to bear in mind that PIKE is definitely not an estimate of the rate of poaching – it is simply an estimate of the probability that a carcass encountered by patrols was illegally killed. Some of the estimated site means shown in Figure 3 have rather wide interval estimates, but this is probably a fair reflection of the uncertainties that underlie the estimates. The Bayesian approach ensures that the estimates include not only uncertainty inherent in the data, but also the uncertainty of the model itself (the latter source of uncertainty being frequently ignored in conventional statistical analyses). In spite of these wide intervals, some clear patterns do emerge from the analysis. Some Central African sites have high mean PIKE, although there is considerable variation across the sub-region; a similar statement can be made about West Africa, although an important difference between Central and West Africa is the much smaller elephant populations in West Africa. A high mean PIKE conveys a different message in each case – in Central Africa it implies large numbers of poached elephants, whereas in West Africa, as in certain sites in Eastern Africa and at least one in Asia, it highlights small elephant populations that are particularly vulnerable. The site mean PIKE in Asia and Southern Africa tends to be lower than in other regions.

### Potential biases in PIKE

The definition of PIKE as the ratio of number of illegally killed carcasses to all carcasses encountered may sometimes be biased because of background variation in elephant mortality. PIKE could be biased downwards if the total carcass count is high because of adverse environmental conditions, such as drought. If these conditions cause high mortality while the true poaching rate remains constant, then PIKE will be lower. During CoP15 in 2010 it was pointed out that the Tsavo and Samburu-Laikipia sites in Kenya suffered from severe drought that could account for the drop in PIKE observed between 2008 and 2009. The analysis was re-run after eliminating all data from those two sites and the overall pattern in the trend remained largely unchanged (apart from 2002, when a very large proportion of the data came from Samburu-Laikipia). So, in this case at least, the analysis based on PIKE proved to be robust.

In principle, these variations in background mortality could be allowed for in the statistical analysis by a Bayesian hierarchical model in which the number of carcasses encountered by a patrol (the binomial “*n*” in our models) is also considered as a random variable, with, say, a Poisson distribution, and modelled on covariates [24]. However, this analysis would require data at individual patrol level, together with a measure of patrol effort, rather than the site by year aggregated data that we have at present. While such data are available from some MIKE sites, many more sites are hampered by logistical and organisational difficulties, although we anticipate that these problems will be resolved in the near future.

Another source of bias inherent in the definition of PIKE is the implicit assumption that the probability of detection of a carcass is the same for all elephants, illegally killed or not. This assumption is questionable, especially in circumstances where patrols act on intelligence that directs them to illegally killed elephants. This is another source of variation that could be accommodated in the models mentioned above – by explicitly modeling the detection probability, with covariates of its own. On the other hand, if it could be assumed that the detection bias is more or less constant over time, then our estimated trend would still be reliable. However, between site comparisons remain questionable as detection bias is not expected to be the same at all sites. We note also that site year combinations where no carcasses were recorded may be due to low detection probabilities

A data quality issue arises from the conclusions from the present analysis that countries with high PIKE values tend to be those with poor governance and development indicators. The problem is that it is likely that these same factors cause MIKE data to be incomplete or otherwise deficient. It is not clear whether the result is a bias in PIKE, or an estimate with lower precision, or both. If there were under-reporting of illegal killing, then PIKE would be biased downwards, but if detection or reporting of all carcasses was generally deficient then we would expect lower precision in PIKE estimates.

### Conclusions

MIKE is an ambitious project in that it aims to collect standardized data from sites across the entire elephant range, with all of its diversity in resources and capacity. It is perhaps not surprising that the flow of data through the MIKE process has been patchy and sometimes painfully slow. Although the available data has limitations, our analysis achieves the following:

1. estimation of the overall trend in illegal killing;
2. the identification of key drivers of illegal killing of elephants at site and national levels;
3. identification of sites of particular concern;
4. an analytical approach that (i) takes proper account of covariates at different levels in the data hierarchy, and (ii) enables predictions across all sites, including those with little data.

A full causal analysis of all potential drivers of illegal killing including the impact of CITES policy and demand for ivory requires more detailed data. One aspect of data from anti-poaching patrols that has been generally overlooked (here and elsewhere) is that the patrols are not passive observers of the process being monitored – they represent an intervention in that process by exerting a deterrent effect [25]. To account for this a dynamic model is required that uses data at the level of individual patrols rather than the site by year aggregates that we have analysed here. For this modeling approach to be effective it would be imperative to include time-varying covariates at site and country levels, at least for key variables that are likely to influence the trend. It would not be possible to justify fixing the levels of covariates at their 2007 levels, as we have done in the present analysis, (a) for data spanning a greater time period and (b) for modeling dynamic effects. This dynamic modeling approach will also allow the inclusion of demand for ivory as a driver of illegal killing and the potential to consider possible impacts of CITES policy. The natural source of data on demand would be MIKE's partner ETIS which monitors the illicit trade in ivory, leading in a natural way to a combined MIKE-ETIS analysis.

In the meantime, our analysis represents the first attempt at a rigorous analysis of data on the illegal killing of elephants across the entire elephant range and identification of factors that contribute to a causal analysis. The results will be of relevance to the CITES process, not only with immediate consequences, but also as a foundation for further work.

## Supporting Information

Figure S1 Map of Africa sites with site codes.
(TIF)

Figure S2 Map of (A) South Asia and (B) South-East Asia sites with site codes.
(TIF)

Figure S3 **Relationships between country-level variables.** Principal component loading plot from the PCA of the country-level variables. Country codes can be found in Table S1.
(TIF)

Figure S4 **Relationships between site-level variables.** Principal component loading plot from the PCA of the site-level variables. Site codes can be found in Table S1.
(TIF)

Figure S5 **Site-level random effects.** The points are the median estimated values of *u_jk_*, for each site, and the line segments are 95% credible intervals. The numbers are the total numbers of carcasses encountered at the site. The random effects are measured on the logit scale.
(TIF)

Figure S6 **Country-level random effects.** The points are the median estimated values of *v_k_*, for each country, and the line segments are 95% credible intervals. The numbers are the total numbers of carcasses encountered in the country. The random effects are measured on the logit scale.
(TIF)

Text S1 **Details of site-level covariates.**
(DOC)

Text S2 **Rationale for a Bayesian Hierarchical Modeling approach.**
(DOC)

Text S3 **Results of Principal Components Analyses.**
(DOC)

Table S1 **Carcass count data.** The number of illegally killed carcasses (total number of carcasses) found at each site in each year. Site and country codes are provided.
(XLS)

Table S2 **Site data.** Site-level covariates for each site.
(XLS)

Table S3 **Country data.** Country-level covariates for each site.
(XLS)
