# Supplementary figures and images for: Global Trends and Factors Associated with the Illegal Killing of Elephants: A Hierarchical Bayesian Analysis of Carcass Encounter Data
Source: PLoS One. 2011 Sep 2;6(9):e24165. doi: 10.1371/journal.pone.0024165 (PMC3166301; doi:10.1371/journal.pone.0024165)

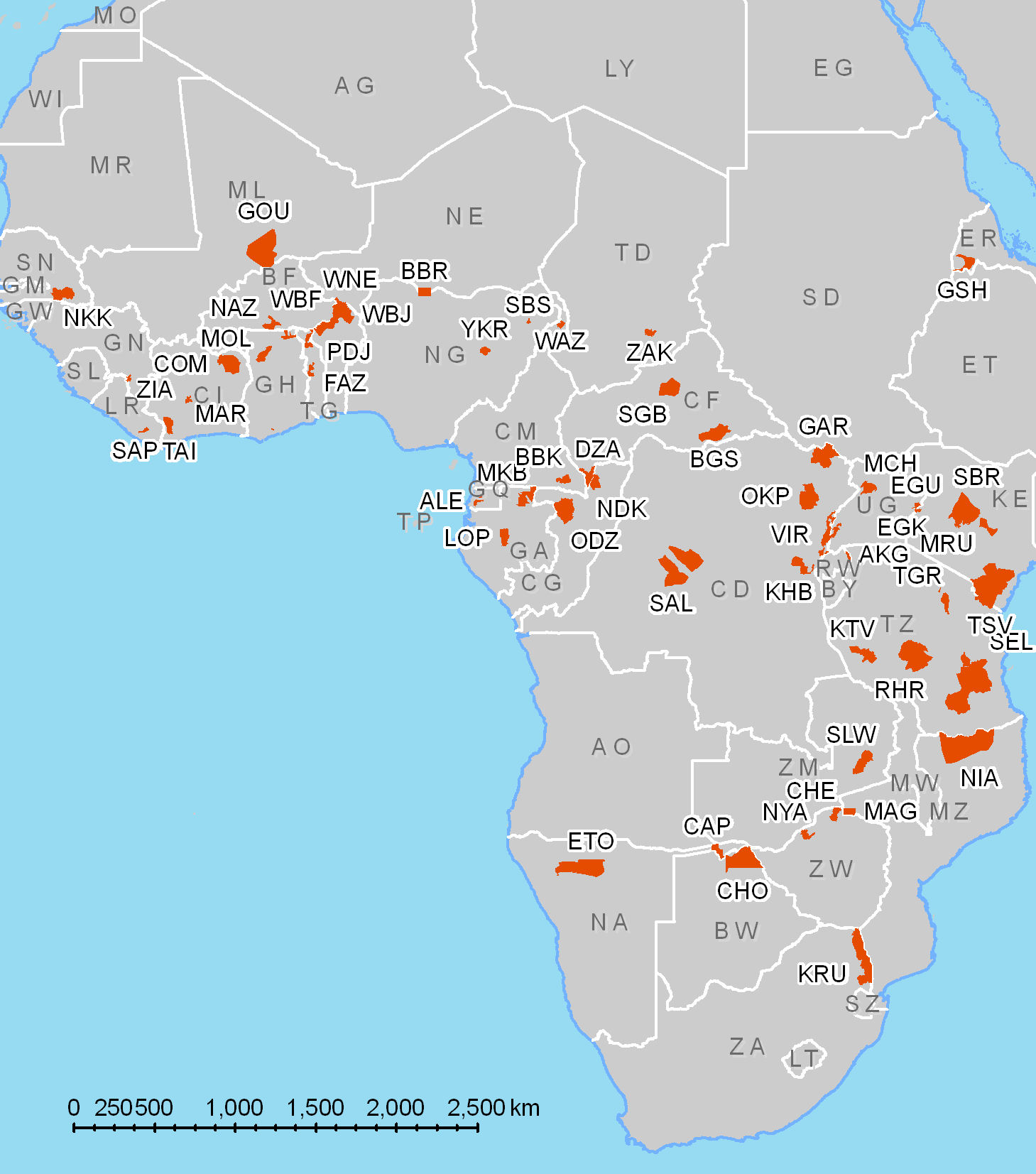

Supplement: Figure S1 — Map of Africa sites with site codes. (TIF) [file pone.0024165.s001.tif]

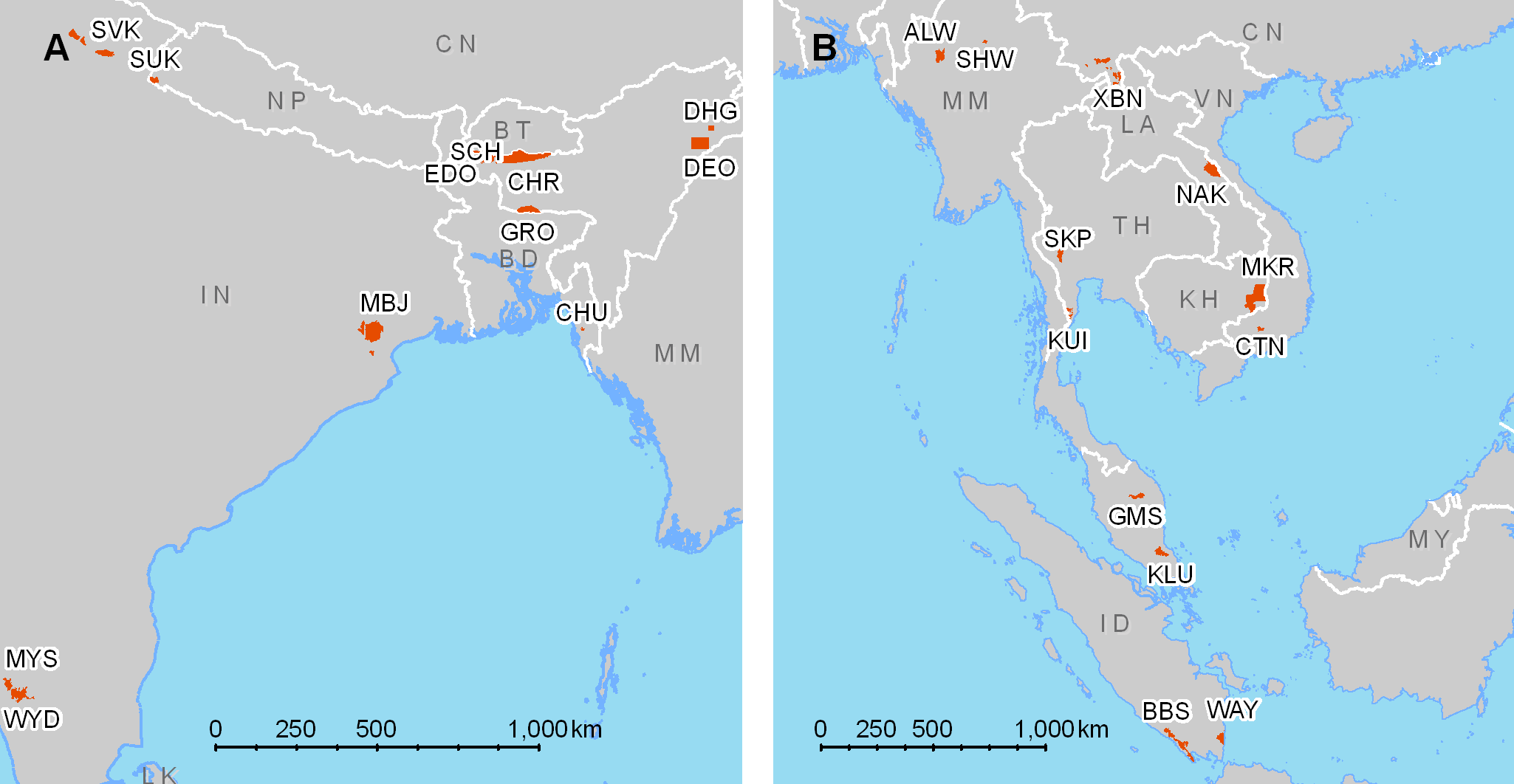

Supplement: Figure S2 — Map of (A) South Asia and (B) South-East Asia sites with site codes. (TIF) [file pone.0024165.s002.tif]

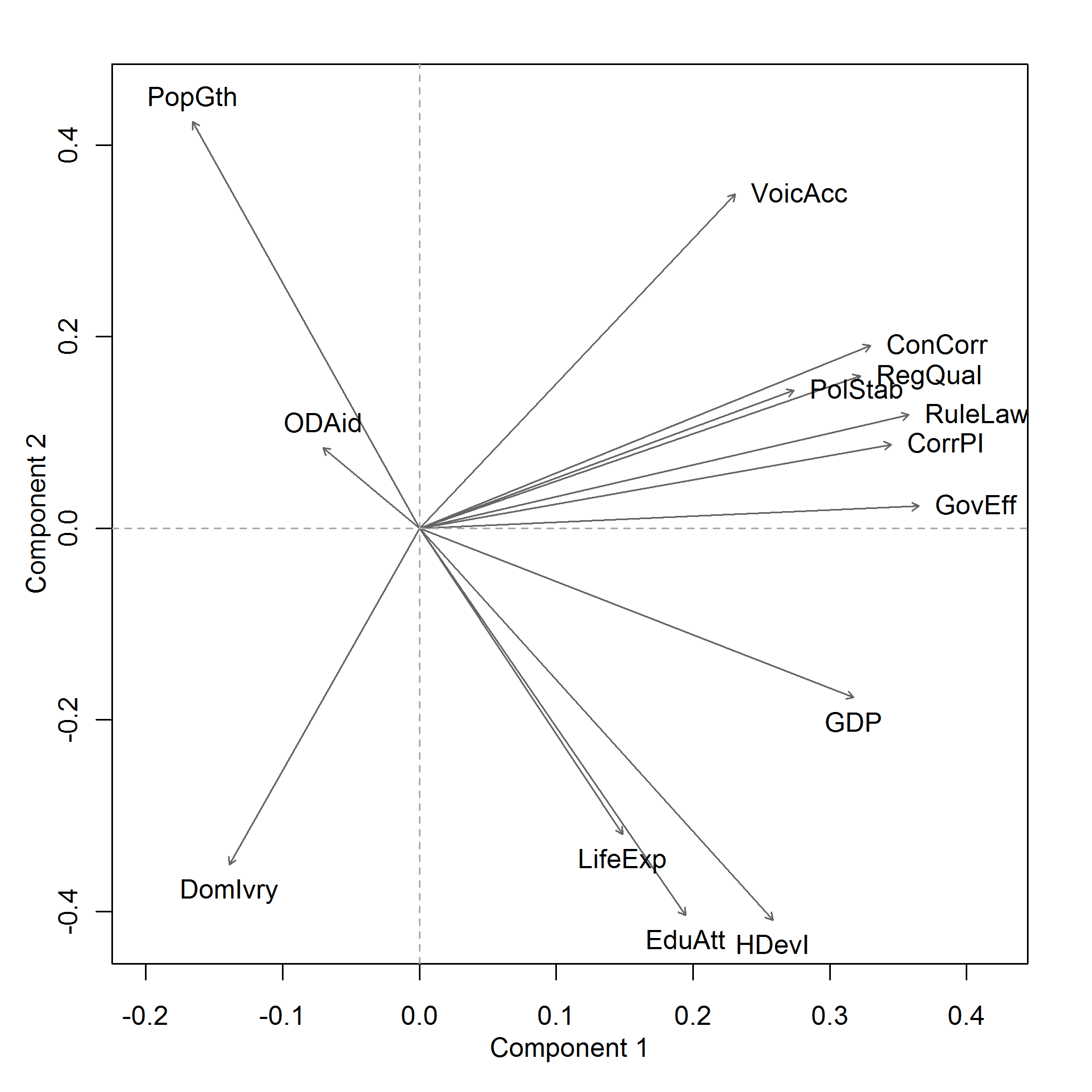

Supplement: Figure S3 — Relationships between country-level variables. Principal component loading plot from the PCA of the country-level variables. Country codes can be found in Table S1. (TIF) [file pone.0024165.s003.tif]

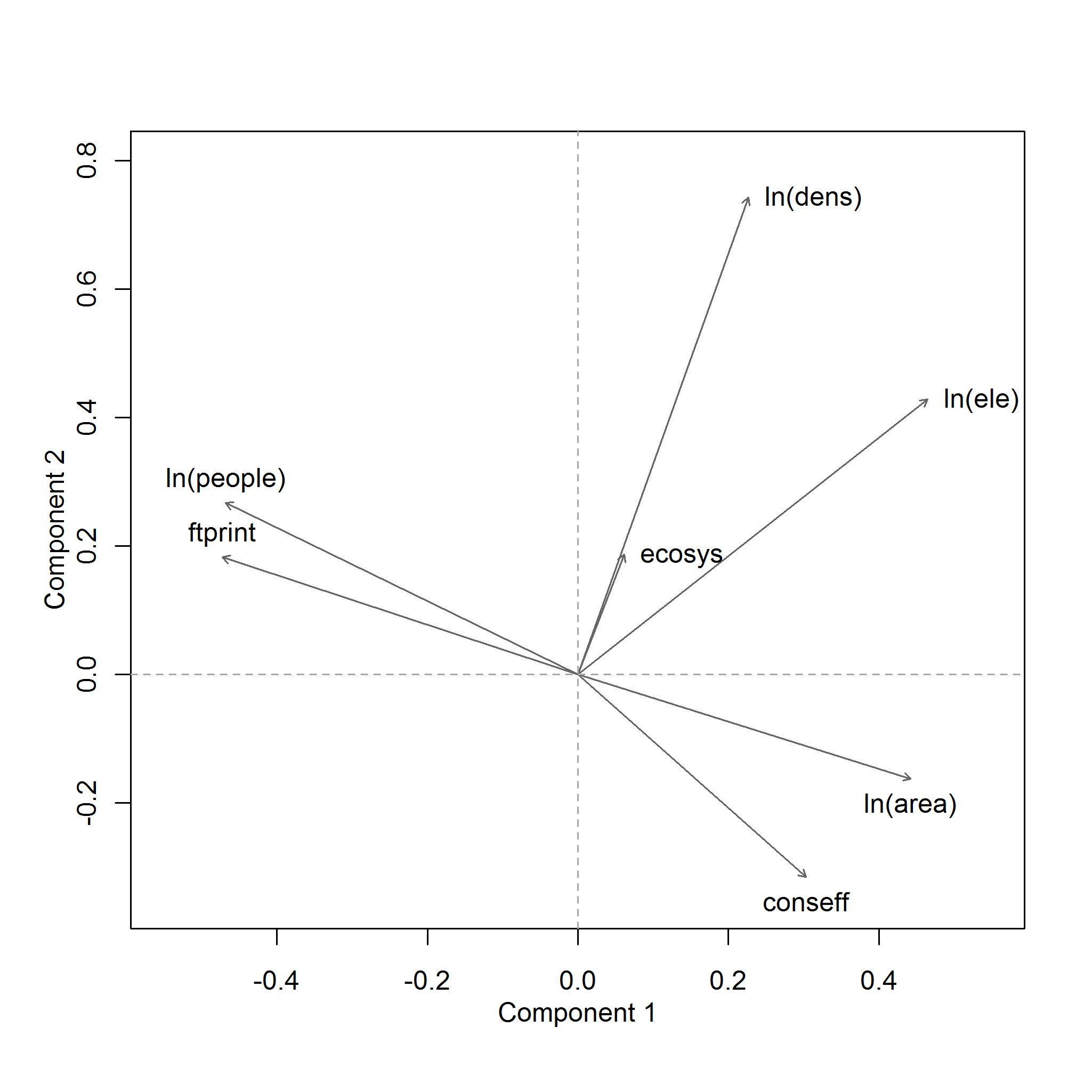

Supplement: Figure S4 — Relationships between site-level variables. Principal component loading plot from the PCA of the site-level variables. Site codes can be found in Table S1. (TIF) [file pone.0024165.s004.tif]

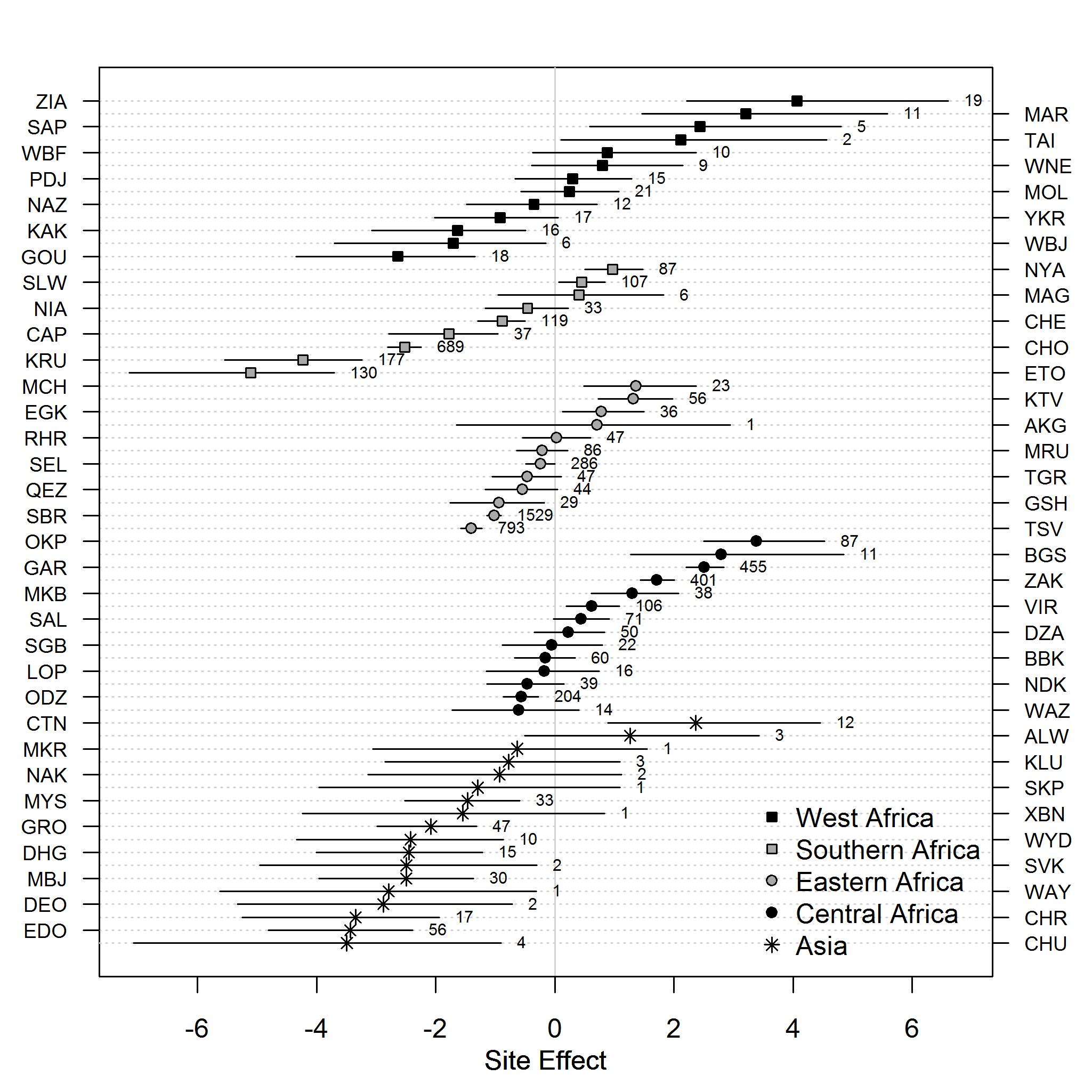

Supplement: Figure S5 — Site-level random effects. The points are the median estimated values of ujk, for each site, and the line segments are 95% credible intervals. The numbers are the total numbers of carcasses encountered at the site. The random effects are measured on the logit scale. (TIF) [file pone.0024165.s005.tif]

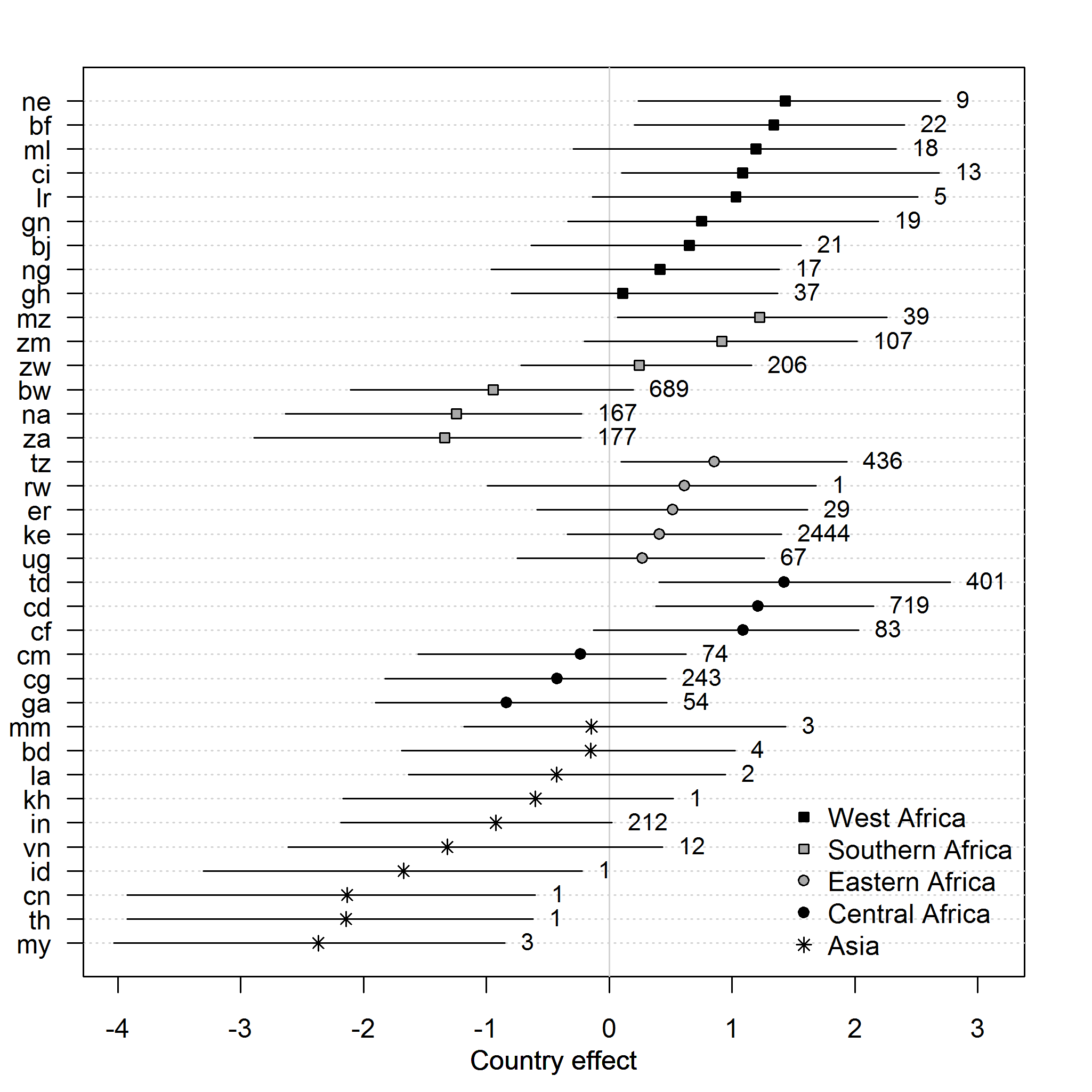

Supplement: Figure S6 — Country-level random effects. The points are the median estimated values of vk, for each country, and the line segments are 95% credible intervals. The numbers are the total numbers of carcasses encountered in the country. The random effects are measured on the logit scale. (TIF) [file pone.0024165.s006.tif]
