# Supplementary material for: Global Trends and Factors Associated with the Illegal Killing of Elephants: A Hierarchical Bayesian Analysis of Carcass Encounter Data
Source: PLoS One. 2011 Sep 2;6(9):e24165. doi: 10.1371/journal.pone.0024165 (PMC3166301; doi:10.1371/journal.pone.0024165)
Supplement: Text S1 — Details of site-level covariates. (DOC) [file pone.0024165.s007.doc]

## Global Trends and Factors Associated with the Illegal Killing of Elephants: a Hierarchical Bayesian Analysis of Carcass Encounter Data

**Robert W. Burn, Fiona M. Underwood, Julian Blanc**

### Supporting Information: Text S1

### Text S1: Details of site-level covariates

For most sites, area of the site and its estimated elephant population (variables *area* and *ele*, respectively) were obtained from the African Elephant Status Report 2007 [1]. More recent survey estimates and Asian estimates were directly obtained from range State Governments and MIKE-sponsored surveys. Rather than classifying of ecosystem type as forest or savannah, we used a more refined, continuous variable, the net primary production (*ecosys*) at the site, defined as the net amount of solar energy converted to plant organic matter through photosynthesis (in units of elemental carbon). Higher values indicate greater vegetative cover. Data for this variable were obtained by overlaying MIKE site boundaries with the spatial data set developed by Imhoff et al [2] and calculating the average values of net primary production therein.

The Human Footprint (Version 2) dataset produced by the Wildlife Conservation Society (WCS) and the Center for International Earth Science Information Network (CIESIN) [3,4] was used to provide a measure of ease of human access and land use type. The Human Footprint is a normalized spatial dataset created from nine global data layers covering human population pressure (population density and population settlements), human land use and infrastructure (built up areas, night-time lights, land use/land cover), and human access (coastlines, roads, railroads, navigable rivers). This variable (*ftprint*) can therefore be considered as encompassing the factors associated with human presence. A variable (*people*) representing human population density was derived from the LandScan ™ dataset (<http://www.ornl.gov/sci/landscan/>). This comprises a worldwide population database compiled on a 30" by 30" latitude/longitude grid (which equates to an approximate 1km resolution). Census counts (at sub-national level) are apportioned to each grid cell based on likelihood coefficients, which are based on proximity to roads, slope, land cover, night time lights, and other information. Data for human footprint and population density were obtained by overlaying the source datasets with boundaries comprising the MIKE sites and a 20 km buffer around each one of them. The values used for analysis were calculated as the average value of all the grid cells contained within these boundaries.

### References

1. Blanc JJ, Barnes RFW, Craig GC, Dublin HT, Thouless CR, Douglas-Hamilton I, Hart JA (2007). *African Elephant Status Report 2007*. Gland: IUCN.
2. Imhoff ML, Bounoua L, Ricketts T, Loucks C, Harriss R, Lawrence WT (2004). Global patterns in human consumption of net primary production. *Nature* 429 870--873
3. Wildlife Conservation (WCS) and Center for International Earth Science Information Network (CIESIN) (2005). *Last of the Wild Data Version 2, 2005 (LWP-2): Global Human Footprint data set (HF)*.
4. Sanderson EW, Jaiteh M, Levy MA, Redford KH, Wannebo AW, Woolmer G (2002). The Human Footprint and the Last of the Wild. *BioScience* 52, no. 10: 891.
