# Supplementary material for: Global Trends and Factors Associated with the Illegal Killing of Elephants: A Hierarchical Bayesian Analysis of Carcass Encounter Data
Source: PLoS One. 2011 Sep 2;6(9):e24165. doi: 10.1371/journal.pone.0024165 (PMC3166301; doi:10.1371/journal.pone.0024165)
Supplement: Text S2 — Rationale for a Bayesian Hierarchical Modeling approach. (DOC) [file pone.0024165.s008.doc]

## Global Trends and Factors Associated with the Illegal Killing of Elephants: a Hierarchical Bayesian Analysis of Carcass Encounter Data

**Robert W. Burn, Fiona M. Underwood, Julian Blanc**

### Supporting Information

### Text S2: Rationale for a Bayesian Hierarchical Modeling approach

Although adding complexity to the analysis, using hierarchical models is important for two reasons. First, ignoring the multilevel structure amounts to assuming that all observations are mutually uncorrelated with each other, both within and between sites and countries. This is a strong assumption and is unlikely to be even approximately true: observations at the same site are likely to be more similar than observations from different sites. The second reason is that in a conventional single level generalized linear model that includes a site-level explanatory variable, it is implicitly assumed that *all* of the site variation is accounted for by that variable, thus increasing the chance of inferring a significant association when in fact there is none. In a hierarchical model, mean differences between sites (and countries) are accounted for by the random effects, and if a site-level (or country-level) explanatory variable turns out to be significant then the effect can be interpreted as additional to the overall site (or country) mean differences.

An added bonus in a hierarchical modeling approach is that predictions can be obtained for all sites in the analysis, even those where only a few carcasses are observed. This is because hierarchical modeling enables a certain amount of sharing, or pooling, of information across sites and countries, an effect known as “shrinkage” [1]. The effect of this is that a mean at a site with few observations is a weighted mean of observations at that site together with observations from other, related sites.

Because of known difficulties with testing fixed effects [1], and also to take full account of all sources of uncertainty in the data, models for estimating the effects of site- and country-level covariates were fitted in a Bayesian framework, using Markov Chain Monte Carlo (MCMC) [2] for model fitting, with non-informative priors for all model parameters. The Bayesian approach ensures that the estimates include not only uncertainty inherent in the data, but also the uncertainty of the model itself (the latter source of uncertainty being frequently ignored in conventional statistical analyses). Statistical computations were performed using the R software (<http://www.r-project.org/>), while WinBUGS (<http://www.mrc-bsu.cam.ac.uk/bugs/winbugs/>) was used for the Bayesian computations.

### References

1. Gelman A and Hill J (2007). *Data Analysis using Regression and Multilevel/Hierarchical Models*. Cambridge UP.

2. Gelman A, Carlin JB, Stern H, Rubin DB (2003). *Bayesian Data Analysis*. London: CRC Press.
