# Supplementary material for: Global Trends and Factors Associated with the Illegal Killing of Elephants: A Hierarchical Bayesian Analysis of Carcass Encounter Data
Source: PLoS One. 2011 Sep 2;6(9):e24165. doi: 10.1371/journal.pone.0024165 (PMC3166301; doi:10.1371/journal.pone.0024165)
Supplement: Text S3 — Results of Principal Components Analyses. (DOC) [file pone.0024165.s009.doc]

## Global Trends and Factors Associated with the Illegal Killing of Elephants: a Hierarchical Bayesian Analysis of Carcass Encounter Data

**Robert W. Burn, Fiona M. Underwood, Julian Blanc**

### Supporting Information

### Text S3: Results of the PCA Analyses

The first two components of the PCA for country-level variables accounted for 70.8% of the total variation (50.8% for the first and 20.0% for the second). A plot of the first two principal component loadings, (Figure S3) shows clear groupings of variables. First, the World Governance Indicators *ConCorr, RegQual, PolStab, RuleLaw and GovEff,* together with Transparency International’s *CorrPI* form a group with *VoicAcc* somewhat less strongly associated. The next obvious group is *HDevI, EduAtt, LifeExp* and *PopGth* (the latter negatively associated). It is not surprising that *EduAtt* and *LifeExp* cluster together with *HDevI*, given that the latter is a composite index incorporating the others. These two groups appear to represent *governance* and *development*. Although distinct, the plot also indicates a degree of correlation between the two groups. The economic indicator *GDP* seems to be, together with *ODAid,* partially associated with both of these groups (*ODAid* being strongly negatively correlated with *GDP*). The domestic ivory market score, *DomIvry*, seems to be negatively associated with governance, suggesting that countries with good governance have smaller domestic ivory markets.

The first two components of a PCA of the seven site variables accounted for 63.9% of the total variation (44.9% for the first component and 19.0% for the second), and the resulting principal component loading plot is shown in Figure S4. Note that *ftprint* and ln(*people*) appear to be strongly correlated and that *area* is negatively correlated with both of them. It turns out that most of the *ecosys* effect is taken up by the third principal component, which accounts for 18.7% of the variation, and therefore appears quite weak in the plot of the first two components. This fact could also explain why, in the plot, *ecosys* appears to be highly correlated with ln(*dens*), whereas in fact there is no correlation. There is little association between conservation effort *conseff* and elephant population density ln(*dens*) or numbers ln(*ele*), but there appears to be some degree of association between *conseff* and the size of the site ln(*area*).
